# Is inflammaging an auto[innate]immunity subclinical syndrome?

**DOI:** 10.1186/1742-4933-3-12

**Published:** 2006-12-16

**Authors:** Sergio Giunta

**Affiliations:** 1 Clinical Laboratory & Molecular Diagnostics, Geriatric Hospital INRCA-IRCCS, Via Montagnola 81-60100, Ancona, Italy

## Abstract

The low-grade, chronic, systemic inflammatory state that characterizes the aging process (inflammaging) results from late evolutive-based expression of the innate immune system. Inflammaging is characterized by the complex set of five conditions which can be described as 1. low-grade, 2. controlled, 3. asymptomatic, 4. chronic, 5. systemic, inflammatory state, and fits with the antagonistic pleiotropy theory on the evolution of aging postulating that senescence is the late deleterious effect of genes (pro-inflammatory versus anti-inflammatory)that are beneficial in early life. Evolutionary programming of the innate immune system may act via selection on these genetic traits. Here I propose that the already acquired knowledge in this field may pave the way to a new chapter in the pathophysiology of autoimmunity: the auto-innate-immunity syndromes. Indeed, differently from the well known chapter of conventional autoimmune diseases and syndromes where the main actor is the adaptive immunity, inflammaging may constitute the subclinical paradigm of a new chapter of autoimmunity, namely that arising from an autoimmune inflammatory response of the innate-immune-system, an old actor of immunity and yet a new actor of autoimmunity, also acting as a major determinant of elderly frailty and age-associated diseases.

## Auto [innate]immunity: a new chapter of autoimmunity

The term "Inflammaging" has been coined by Claudio Franceschi to explain the now widely accepted phenomenon that ageing is accompanied by a low-grade chronic, systemic up-regulation of the inflammatory response and that the underlining inflammatory changes are also common to most age-associated diseases [1-8].

From a review of the literature on Inflammaging [1-8] I suggest that, like acute inflammation is well known to be associated with the five cardinal features 1. rubor (redness), 2. tumor (swelling), 3. dolor (pain), 4. calor (warm), 5. functio lesa (loss of function), on the contrary, inflammaging is characterized by the complex set of five conditions which can be described as 1. low-grade, 2. controlled, 3. asymptomatic, 4. chronic, 5. systemic, inflammatory state.

The inflammatory scenario that characterizes inflammaging constitutes a highly complex response to various subtle internal and environmental inflammatory stimuli mediated mainly by the increased circulating levels of pro-inflammatory cytokines. Inflammaging also generates Reactive Oxygen Species (ROS) causing both oxidative damage and eliciting an amplification of the cytokines' release, thus perpetuating a vicious cycle resulting in a chronic systemic pro-inflammatory state where tissue injury and healing mechanisms proceed simultaneously and damage slowly accumulates asymptomatically over decades and is a major determinant both of the ageing process and of the development of age-associated diseases [3,5-8].

Moreover, Claudio Franceschi, Tom Kirkwood, and Calogero Caruso [1,8,9] as well as other authors, postulate that both the ageing process and age-associated diseases are late consequences of evolutionary programming for a pro-inflammatory response mainly selected to resist infections and for a successful response to wound healing in early age, a view that has been discussed in the light of the antagonistic pleiotropy theory [1,8,9]. Such a theory on the evolution of aging postulates that senescence is the late deleterious effect of genes that are beneficial in early life. Evolutionary programming of the innate immune system may act via selection on these genetic traits.

Inflammaging is triggered by the first line of biological defense i.e. the innate immunity that operates by detection of a broad range of injuries inducing the activation of inflammatory responses [1,4,7]. The mononuclear phagocytes lineage plays a pivotal role in innate immunity that does not require clonal expansion of cell populations [10]. Moreover, several other cell types contribute to innate immunity by expressing pattern recognition receptors, namely various scavenger and Tolllike receptors [11]. These cell encoded proteins recognize ligands from damaged tissues and induce host responses by transmembrane signals that activate NF-kB and mitogen dependent protein kinase pathway [12]. Toll-like receptor activation also induces the expression of a wide variety of number of genes encoding proteins, such as cytokines, with regulatory functions upon cell activations and tissue inflammation [13].

Here I propose that the already acquired knoledge in this field may pave the way to a new chapter in the pathophysiology of autoimmunity: the auto [innate]immunity syndromes, where Inflammaging may constitute the subclinical paradigm of such a new chapter of autoimmunity.

Moreover, while the innate immunity induced Inflammaging may remain sub-clinical as a determinant of the ageing process, however, upon the presence of high responder inflammatory genotypes (pro-inflammatory cytokines versus anti-inflammatory cytokines), this auto [innate]immunity syndrome may determine or at least contribute to age-associated frailty and chronic diseases including cardiovascular diseases, Alzheimer's disease, insulin-resistance and diabetes, osteoarthritis, osteoporosis, sarcopenia. Therefore, differently from the well known chapter of conventional autoimmune diseases and syndromes where the main actor is the adaptive immunity, Inflammaging may constitute the subclinical paradigm of a new chapter of autoimmunity, namely that arising from an auto-immune inflammatory response of the innate-immune-system an old actor of immunity and yet a new actor of autoimmunity.
